# Novel Platform Development Using an Assembly of Carbon Nanotube, Nanogold and Immobilized RNA Capture Element towards Rapid, Selective Sensing of Bacteria

**DOI:** 10.3390/s120608135

**Published:** 2012-06-12

**Authors:** Elizabeth I. Maurer, Kristen K. Comfort, Saber M. Hussain, John J. Schlager, Sharmila M. Mukhopadhyay

**Affiliations:** 1 Molecular Bioeffects Branch, Human Effectiveness Directorate AFRL/RHDJ, Wright Patterson Air Force Base, Dayton, OH 45433, USA; E-Mails: elizabeth.maurer.ctr@wpafb.af.mil (E.I.M.); kristen.comfort.ctr@wpafb.af.mil (K.K.C.); john.schlager@wpafb.af.mil (J.J.S.); 2 Center for Nanoscale Multifunctional Materials, Wright State University, Dayton, OH 45435, USA; E-Mail: sharmila.mukhopadhyay@wright.edu

**Keywords:** biosensor, gold nanoparticle, carbon nanotube, nanomaterial, aptamer

## Abstract

This study examines the creation of a nano-featured biosensor platform designed for the rapid and selective detection of the bacterium *Escherichia coli*. The foundation of this sensor is carbon nanotubes decorated with gold nanoparticles that are modified with a specific, surface adherent ribonucleiuc acid (RNA) sequence element. The multi-step sensor assembly was accomplished by growing carbon nanotubes on a graphite substrate, the direct synthesis of gold nanoparticles on the nanotube surface, and the attachment of thiolated RNA to the bound nanoparticles. The application of the compounded nano-materials for sensor development has the distinct advantage of retaining the electrical behavior property of carbon nanotubes and, through the gold nanoparticles, incorporating an increased surface area for additional analyte attachment sites, thus increasing sensitivity. We successfully demonstrated that the coating of gold nanoparticles with a selective RNA sequence increased the capture of *E. coli* by 189% when compared to uncoated particles. The approach to sensor formation detailed in this study illustrates the great potential of unique composite structures in the development of a multi-array, electrochemical sensor for the fast and sensitive detection of pathogens.

## 1. Introduction

By definition, a biosensor is a self-contained integrated device that is capable of providing specific quantitative or semi-quantitative information using a biological recognition element, which retains direct spatial contact with an electrochemical transduction element [1]. In an effort to achieve highly targeted and diverse analyte detection, numerous material platforms are currently being explored for their potential in sensing systems. However, due to their established electrical transmission properties [2] and the ability for direct conjugation with other molecules, carbon nanotubes (CNTs) have recently gained significant attention in the biosensors community. CNTs are also distinctive in the respect that the majority of their atoms reside on or near the particle surface, with the consequence that molecular modifications have a significant impact on its electrical properties [3]. An additional advantage of nanotubes is that owing to their high surface area to volume characteristic, they inherently possess a greater number of available binding sites for chemicals, biomolecules, and even other nanoparticles. With the addition of this increased surface area available for target attachment, an increased sensitivity would be expected [4,5]. The modification of CNT surface chemistry has been previously demonstrated for uses in novel electronics, filtration media, biomedical devices, and biosensors [6,7].

As the field of sensor technology has developed, the need for a whole-cell, real-time mechanism for the detection of specific bacteria has emerged. In 2010 alone, according to the Centers for Disease Control (CDC) and Prevention, over 70,000 *E. coli* and 40,000 *Salmonella* infection cases were reported; demonstrating a clear justification for developing a means of pathogen detection. As current pathogen detection and identification procedures require approximately 48 hours, there is a critical need for earlier detection of pathogens, before they can rapidly spread and induce harmful effects. Using Fourier Transform Infrared Spectroscopy, a new technique has been developed to detect the presence of *E. coli* within 6 hours [8]. While this is a vast improvement, it does not even approach the known 20 minute doubling time of *E. coli*. Breakthrough advances are needed to further reduce these detection windows to an appropriate microbial scale, and expand sensor capabilities to include additional classes and orders of pathogens.

One such technological advancement is the utilization of cell specific molecular recognition systems for detection, a field that has been rapidly evolving. Previous studies have successfully demonstrated increased identification of *Salmonella* [9] and *E. coli* O157:H7 strains [10,11] through the use of specific antibody-based detection systems. However, innate problems are associated with the use of antibodies, including the need for an *in vivo* system for generation and their restriction to only working under physiological conditions. Recently, aptamers have emerged as a more advantageous target recognition agent when compared to antibodies [12]. Aptamers, short oligonucleotide sequences, have proven to have a higher degree of specificity, be more biologically stable [13] and have the benefit of being easily tailored to innumerable targets through a variety of attachment mechanisms [14].

**Figure 1.**
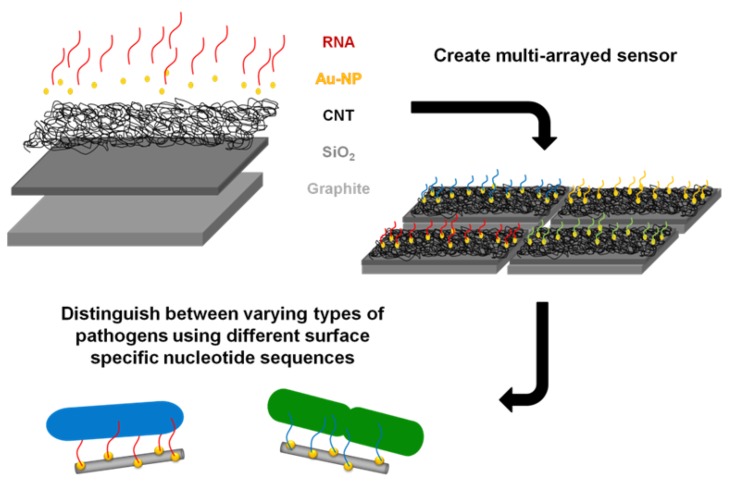
Schematic depicting each component/layer of the designed biosensor as well as illustrating the potential application of the construction of a multi-arrayed sensor.

The goal of this study was to successfully conjugate a pathogen specific aptamer with a novel base material in an effort to develop a unique bacterium targeted biosensor. A platform base of CNTs was selected as they have previously been utilized in electrochemical sensor formation for biomolecules [15], due to their structural-dependent enhanced electrical properties. The enhanced surface area of CNTs readily allows for association with other nanomaterials, which further increases available attachment sites and augments sensitivity. For example, a previous study attached nanocubes to CNTs which improved the binding and effectiveness of a nanocatalyst [16]. The layout of this study's proposed biosensor is illustrated in Figure 1 and is supported by with a graphite foundation coated with a thin silica layer. CNTs were then grown at high density upon this base through a deposition method. Gold nanoparticles were directly synthesized onto the CNT surface to simultaneous increase exposed surface area and provide an adsorption location for the synthetic application of a ribonucleic acid (RNA) sequence. This RNA sequence was designed to provide unique binding specificity to the surface of the DH5α *E. coli* strain, as demonstrated by So and colleagues [17]. This study verified the successful capture of *E. coli* to the RNA coated gold nanoparticles with high affinity and laid the groundwork for future biosensor development from this established composite model. Furthermore, the inclusion of additional capture elements targeted to sense different pathogens could easily be integrated to produce a multi-arrayed sensor.

## 2. Experimental Section

### 2.1. Cell Culture

Unless otherwise noted all chemicals were purchased from Fisher Scientific. DH5α *E. coli* bacterial strain was obtained from ATCC. Cultures were inoculated by the addition of 10 μL of frozen stock solution to 6 mL of sterile Luria Bertani (LB) broth, comprised of 20 g of powder dissolved in 1 L of water. The cells were then incubated overnight at 37 °C in a humidified 5% CO_2_ atmosphere. The next day, the cells were washed by centrifugation and re-suspended in phosphate buffered saline (PBS) at a final concentration of 10^6^ cells/mL.

### 2.2. Carbon Nanotube Growth

Highly oriented pyrolitic graphite sheets (Molecular Imaging) were cut and sanded into rectangles to serve as the platform base. A nano-hair type layer of strongly grafted CNT was fabricated on these surfaces through a two-step process [18] and used to create a hierarchical structure. In brief, this method involves activation of the graphite surface with plasma-derived silica, followed by Chemical Vapor Deposition (CVD) growth of CNT using a xylene-ferrocene solution and hydrogen in a two-stage furnace. To prevent oxidation at high temperature, argon was applied during the entire growth process. Verification of CNT growth was confirmed with scanning electron microscopy (SEM).

### 2.3. Gold Nanoparticle Attachment

The resultant CNT/graphite composites were cut into smaller pieces and adhered to the side of a beaker using double sided carbon tape. This allowed for the CNTs to be fully immersed within the solution without risk of contact and damage by the magnetic stir bar. For gold nanoparticle synthesis, 43.75 mL of water was stirred for 3 minutes followed by the addition of 3 mL of a 0.4% HAuCl_4_ (MP Biomedicals LCC) solution and an additional minute of stirring. Next, 5 mL of 0.1% sodium citrate (Na_3_C_6_H_5_O_7_) solution and 0.01% sodium borohydride (NaBH_4_) were introduced, which instantly turned the solution from pale yellow to maroon. This solution was continuously stirred for 2 minutes to allow freshly synthesized NPs on CNTs to stabilize. The solution was then removed and the samples, still attached to the side of the beaker, were rinsed with distilled water. After washing and air drying, the samples underwent SEM and energy dispersive spectroscopy (EDS) spectra analysis to verify gold nanoparticle synthesis.

### 2.4. DNA Transcription

The surface-specific RNA sequence used was based off of a sequence discovered by So and colleagues [17] using a systematic evolution of ligands by exponential enrichment (SELEX) process. To obtain the RNA needed for this study, DNA transcription was carried out using the following sequences in accordance with the manufacturer's instructions.

SS-DNA: 5′ - **TAT AGT GAG TCG TAT TA** – 3′

LS-DNA: 5′ - **TAA TAC GAC TCA CTA TA**G GGA GA GGG AGA GCG GAA GCG TGC TGG GTC GCA GTT TGC GCG CGT TCC AAG TTC TCT CAT CAC GGA ATA CAT AAC CCA GAG GTC GAT GGG GGG GGG GGG – 3′

In brief, SS-DNA and LS-DNA were combined and annealed at 94 °C for 2 min then transcribed using a Durascribe^®^ T7 Transcription Kit containing 2′-F-UTP and 2′-F-CTP (Epicentre Biotechnologies) at 37 °C for 6 hours and stored at −20 °C to prevent degradation.

After successful DNA transcription, a thiol group was hybridized on the end of the RNA, which facilitated its attachment to the gold nanoparticles. This is due to the high affinity thiol has to gold and was accomplished through a compliment nucleotide hybridization of cytosine and guanine. A 12 cytosine chain containing a thiol group on its end was annealed at 94 °C for 2 minutes to the 12 guanine spacer on the RNA transcribed product sequence. For verification of successful transcription as well as concentration determination of the RNA, absorbance at 260 nm was recorded using a NanoDrop spectrophotometer.

### 2.5. RNA Attachment to Gold Nanoparticles

Prior to introduction on the final platform, RNA was first attached to the colloidal gold nanoparticles in solution. These particles were synthesized using the same process as stated earlier on the carbon nanotube samples. After synthesis, the particle concentration was determined using UV-Vis analysis and the Beer-Lambert Law (A = εcl), where A = absorbance in optical density units, ε = exstinction coefficient (at the surface plasmon wavelength), l = path length through sample, and c = concentration of nanoparticles. (A= obtained from UV-Vis spectrum, ε = 1.935 × 10^10^ M^−1^·cm^−1^, l = 1 cm)

For RNA attachment, 1 mL of the 50–60 nm gold nanoparticle solution was pipetted into a scintillation vial with 1 mL of thiolated RNA, stirred briefly, and incubated in the dark for 20 minutes. Next, 246 μL of 100 mM phosphate buffer and 40 μL of 2M sodium chloride (NaCl) was added to the mixture and stirred for 20 minutes. Additional NaCl (40 μL of 2M) was then added to the solution and stirred for another 20 minutes. This was repeated a final time and left to incubate overnight protected from light. The next day the particles are centrifuged at 12,000 × g for 15 minutes and re-suspended in 100 μL of a 100 mM NaCl and 25 mM tris acetate solution. Lastly, the particles were washed two additional times. This process was conducted on gold nanoparticles without the addition of RNA to serve as a control. To confirm attachment, Dynamic Light Scattering (DLS) and Zeta Potential analyses were conducted on a Malvern Zetasizer.

### 2.6. Binding of E. coli Proof of Concept

After RNA coating, the gold particles were then exposed to *E. coli* to quantify targeted RNA attachment. All experiments were conducted in triplicate to verify findings. First, at room temperature, 400 μL of 10^6^ concentration of DH5α in PBS was combined with 100 μL of the coated particles. This experiment was run in parallel with 100 μL of bare gold nanoparticles to allow for direct comparison. The samples were rocked for 20 minutes then spun down at low speed, to pellet the bacteria but not the unbound particles. The cellular pellet was suspended in sterile water for UV-Vis measurements. One milliliter of the sample is placed in a quartz cuvette and absorption is measured from ∼200 nm to 800 nm. Baseline absorbance spectra from excess RNA and *E coli* were removed from the overall absorption to ensure only readings from the nanoparticles were measured.

## 3. Results and Discussion

### 3.1. Construction of Sensor Platform Base

**Figure 2.**
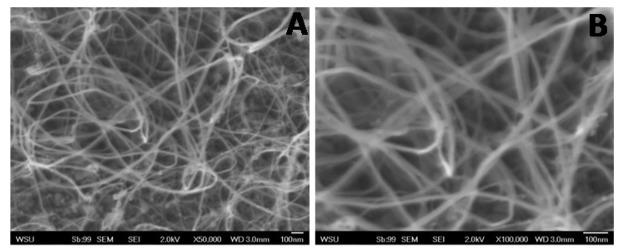
SEM images of CNTs grown on silica following chemical vapor deposition process. The same view was captured at a magnification of 50,000× (**A**) and 100,000× (**B**).

The present study was aimed at developing a novel sensor to detect bacteria based on carbon nanotubes functionalized with gold nanoparticles with a ribonucleic acid (RNA) sequence attached as a capture element. Highly oriented pyrolitic graphite (HOPG) was used as the base carbon structure due to its ability to withstand high temperatures as well as its relative inertness. To confirm CNT growth on the graphite and visualize the packing density SEM imaging was conducted. As seen in Figure 2 CNTs were successfully grown on the surface of silica coated graphite at a high density. Furthermore, minimal amounts of excess iron particles were found on the surface of the nanotubes indicative of the high CNT quality.

**Figure 3.**
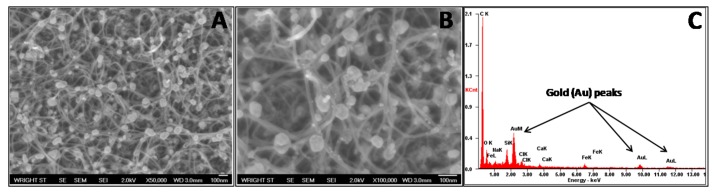
Authentication of gold nanoparticles on the CNT surface was performed with SEM at a magnification of 50,000× (**A**) and 100,000× (**B**). EDS spectra analysis also demonstrated proof of gold existence within the composite samples (**C**).

To ensure the presence of gold nanoparticles, their successful attachment to the CNT surface, and even distribution, SEM images were obtained (Figure 3(A,B)). These images demonstrate well dispersed, strongly adhered gold nanoparticles with a low agglomeration level. For these particles, a size distribution of 44.9 ± 14.4 nm was determined. Furthermore, EDS results indicated a strong presence of gold within the sample, further confirming successful composite formation (Figure 3(C)).

### 3.2. Verification of RNA Attachment

Prior to the introduction of *E. coli*, it had to first be verified that RNA was effectively bound to the surface of the gold nanoparticles. To accomplish this task, the hydrodynamic diameter and the surface charge of the gold nanoparticles was measured both prior to and post RNA introduction, as successful attachment of RNA would alter both characteristics. These results are shown in Table 1 and clearly demonstrate a significant increase in size and surface charge. Taken together this data is indicative of a surface modification, thereby validating the presence of RNA on the gold nanoparticles.

### 3.3. RNA Coated Particle Exposure to E. coli

**Figure 4.**
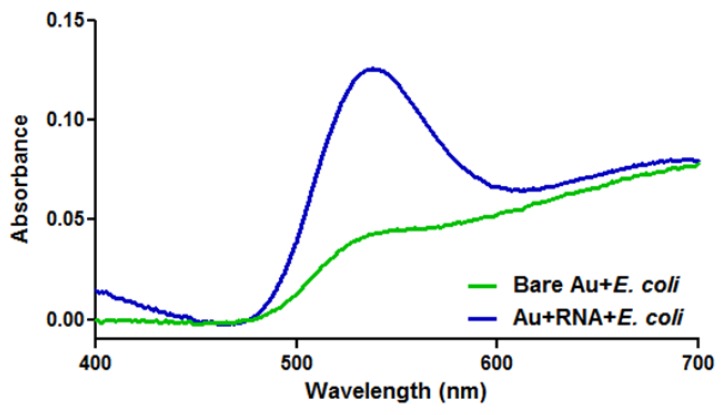
UV-Vis measurements were conducted on the *E. coli* samples containing uncoated and RNA-coated gold nanoparticles. The graph above shows the signature peak of gold nanoparticles at approximately 525 nm with a much higher absorbance and slight peak shift seen with the RNA-coated particles. The increased amount of absorbance confirms enhanced attachment with the addition of *E. coli* specific RNA.

The next step was the proof of concept experimentation to ascertain if *E. coli* preferentially bound to the RNA coated gold nanoparticles over bare ones. This was achieved by isolating the cellular component after co-incubation and analyzing the gold content. A marked increase in gold associated with the RNA coated samples would signify greater coupling and a higher degree of specificity between *E. coli* and the particles. Figure 4 shows the UV-Vis analysis of the recovered cultures and clearly exhibits the signature peak of gold nanoparticles at approximately 525 nm. Furthermore, the sample incubated with RNA-coated particles produced a gold peak with a significantly higher absorbance and a slight shift as compared to the uncoated particles. The increased amount of absorbance is a direct result of a higher concentration of gold nanoparticles present; indicating a greater affinity of RNA functionalized particles to the *E. coli*. When further analyzed, this data confirms a 189% enhanced bacterial binding in the presence of *E. coli* specific RNA versus the uncoated particles; calculated by dividing the difference in peak absorbance by the uncoated absorbance value.

### 3.4. Initial Testing of the Fully Constructed Biosensor

**Figure 5.**
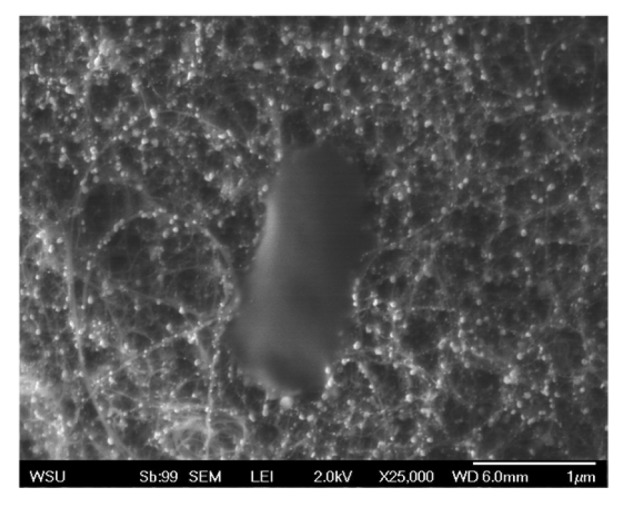
Represenative SEM image of successful *E. coli* capture on the RNA coated gold nanoparticles attached to a carbon nanotube substrate.

After independently confirming gold nanoparticle growth on the bound CNTs and successful RNA coating of gold nanoparticles, the final step was to construct and assess the effectiveness of the complete biosensor. Therefore, following synthesis of gold nanoparticles on the graphite bound CNTs the particles were subsequently functionalized with the thiolated RNA sequence specific for the DH5α *E. coli* strain. Following introduction of *E. coli* to the biosensors, they were washed and SEM imaged to evaluate extent of microbial association. As seen in Figure 5, this sensor was capable of strongly binding *E. coli* molecules, however no significant difference was observed between the bare and RNA coated sensors. After further investigation, we believe that this lack of RNA specificity is due to interactions between the RNA and the carbon nanotube surface. It has been shown that CNTs interact with the conformation of nucleic acids [19] due to their π-π stacking interactions [20]. We speculate that this unintentional coupling is resulting in the folding over of a high percentage of RNA molecules, thus making them unavailable and reducing potential binding sites. Future research will focus on developing a mechanism to minimize this concern and improve targeted *E. coli* binding, as well as an amperometric analysis of the overall structure.

## 4. Conclusions

There exists a strong need within the sensors community to develop the capability for the real-time sensing mechanism for foreign pathogens. The work presented in this study defines a composite bases sensor that incorporates specific RNA sequences to selectively bind a microbial agent. CNTs were successfully grown on a graphite substrate and subsequently served as a point of synthesis for gold nanoparticles. In solution, we successfully attached a thiolated RNA that recognizes *E. coli* sequence to gold nanoparticles. These RNA coated gold nanoparticles were shown to enhance *E. coli* capture by 189% when compared to bare gold nanoparticles. Following full construction of the composite sensor, preliminary tests showed no statistical significance with the addition of RNA, which we suspect is due to interactions occurring between the RNA and CNTs. Even still, we believe this work has great consequence as it lays the ground work for future molecular recognition based sensor constructed from nanomaterials.

Furthermore, this research demonstrated that carbon substrates, in bulk and nano-scale, were able to be utilized in the development of a novel sensor to detect pathogens in the environment. We are confident from this work that utilizing novel nanostructures for the application of a bio-sensor in bacterial detection and identification is attainable. This same sensing approach outlined here can easily be applied to other bacterial surface-specific RNA sequences, attaching them in the same manner, and ultimately creating an electrochemical multi-array sensor to determine the strain of bacteria as well as their aqueous solution concentration. Additionally, this process can be extended to other microorganisms to identify and quantify targets through the development of a hand-held multi-array sensor.

## Figures and Tables

**Table 1.** Characterization of bare and RNA bound gold nanoparticles. The evaluation of the hydrodynamic diameter and the surface charge of these particles was carried out to confirm the presence of RNA functionalization.

**Nanoparticle**	**Size (nm)**	**Surface Charge (mV)**
Bare Au	44.0	−41.8
Au + RNA	55.0	−32.8
